# Lingguizhugan decoction attenuates doxorubicin-induced heart failure in rats by improving TT-SR microstructural remodeling

**DOI:** 10.1186/s12906-019-2771-6

**Published:** 2019-12-11

**Authors:** Xueping Li, Guangmin Xu, Shujun Wei, Baocheng Zhang, Huan Yao, Yuchi Chen, Weiwei Liu, Baojia Wang, Juan Zhao, Yongxiang Gao

**Affiliations:** 1 Hospital of Chengdu University of Traditional Chinese Medicine, Chengdu, 610072 China; 2 Department of Anesthesia, Sichuan Academy of Medical Sciences & Sichuan Provincial People’s Hospital, Chengdu, 610072 China; 3 College of Basic Medicine, Chengdu University of Traditional Chinese Medicine, Chengdu, 611137 China; 4 College of Clinical Medicine, Chengdu University of Traditional Chinese Medicine, Chengdu, 611137 China; 5 College of International Education, Chengdu University of Traditional Chinese Medicine, Chengdu, 610075 China

**Keywords:** Heart failure, Doxorubicin, Lingguizhugan, TT-SR, JP-2, miR-24

## Abstract

**Background:**

Lingguizhugan decoction (LGZG), an ancient Chinese herbal formula, has been used to treat cardiovascular diseases in eastern Asia. We investigated whether LGZG has protective activity and the mechanism underlying its effect in an animal model of heart failure (HF).

**Methods:**

A rat model of HF was established by administering eight intraperitoneal injections of doxorubicin (DOX) (cumulative dose of 16 mg/kg) over a 4-week period. Subsequently, LGZG at 5, 10, and 15 mL/kg/d was administered to the rats intragastrically once daily for 4 weeks. The body weight, heart weight index (HWI), heart weight/tibia length ratio (HW/TL), and serum BNP level were investigated to assess the effect of LGZG on HF. Echocardiography was performed to investigate cardiac function, and H&E staining to visualize myocardial morphology. Myocardial ultrastructure and T-tubule-sarcoplasmic reticulum (TT-SR) junctions were observed by transmission electron microscopy. The JP-2 protein level was determined by Western blotting. The mRNA level of CACNA1S and RyR2 and the microRNA-24 (miR-24) level were assayed by quantitative RT-PCR.

**Results:**

Four weeks after DOX treatment, rats developed cardiac damage and exhibited a significantly increased BNP level compared with the control rats (169.6 ± 29.6 pg/mL versus 80.1 ± 9.8 pg/mL, *P* < 0.001). Conversely, LGZG, especially at the highest dose, markedly reduced the BNP level (93.8 ± 17.9 pg/mL, *P* < 0.001). Rats treated with DOX developed cardiac dysfunction, characterized by a strong decrease in left ventricular ejection fraction compared with the control (58.5 ± 8.7% versus 88.7 ± 4.0%; *P* < 0.001). Digoxin and LGZG improved cardiac dysfunction (79.6 ± 6.1%, 69.2 ± 2.5%, respectively) and preserved the left ventricular ejection fraction (77.9 ± 5.1, and 80.5 ± 4.9, respectively, *P* < 0.01). LGZG also improved the LVEDD, LVESD, and FS and eliminated ventricular hypertrophy, as indicated by decreased HWI and HW/TL ratio. LGZG attenuated morphological abnormalities and mitochondrial damage in the myocardium. In addition, a high dose of LGZG significantly downregulated the expression of miR-24 compared with that in DOX-treated rats (fold change 1.4 versus 3.4, *P* < 0.001), but upregulated the expression of JP-2 and antagonized DOX-induced T-tubule TT-SR microstructural remodeling. These activities improved periodic Ca^2+^ transients and cell contraction, which may underly the beneficial effect of LGZG on HF.

**Conclusions:**

LGZG exerted beneficial effects on DOX-induced HF in rats, which were mediated in part by improved TT-SR microstructural remodeling.

## Background

Heart failure (HF) is a pressing public health issue, and no curative treatment is currently available. Approximately 1–3% of the adult population have been diagnosed with HF, and the lifetime risk of HF is one in five for men and women [1]. In addition, HF is a major and growing problem in most affluent countries because of aging populations and the prolongation of cardiac patients’ lives by modern therapies. Once HF is clinically manifest, the median survival of patients is only 1.7 years for men and 3.2 years for women, with only 25% of men and 38% of women surviving for 5 years after diagnosis [2]. HF has a poor prognosis, and the rate of hospital admission for HF and the associated healthcare costs have dramatically increased recently [3–7]. In the United States, the total medical costs for patients with HF are expected to rise from $20.9 billion in 2012 to $53.1 billion by 2030 [7]. Globally, HF is the leading cause of death due to cardiovascular disease. Although its utility is limited by the availability of donors, cardiac transplantation is the only viable intervention for end-stage HF [8]. Thus, both an understanding of the pathogenesis of HF and the development of novel therapeutic strategies or drugs with improved efficacy are needed.

HF is a complex syndrome caused by structural or functional impairment of ventricular filling or blood ejection. Mechanistically, the hallmarks of HF include abnormal energy metabolism, increased production of reactive oxygen species (ROS), and defects in excitation–contraction (E-C) coupling [9]. Energy insufficiency is a key feature of systolic HF, and mitochondria supply the myocardium with energy [10, 11].

The pace and strength of cardiomyocyte contraction is determined by periodic Ca^2+^ transients, which are regulated by the Ca^2+^-induced Ca^2+^ release (CICR) mechanism. The CICR is located between L-type Ca^2+^ channels (LCCs) in the cell membrane/T-tubules (TTs) and ryanodine receptors (RyRs) in the junctional sarcoplasmic reticulum (SR) [12, 13]. Ca^2+^ release by RyRs is modulated by both global Ca^2+^ transients and local Ca^2+^ release events, which induce aggregation of RyR and LCC into discrete CICR units in the TT-SR junctional structure [14]. During normal physiological signaling, the Ca^2+^ influx through LCCs travels across an approximately 15-nm junctional cleft and activates RyR Ca^2+^ release. Junctophilin-2 (JP-2), a protein that anchors the sarcoplasmic reticulum (SR) to T-tubules (TTs), is a major target for regulation of E-C coupling [15]. MicroRNA-24 (miR-24) has been identified as an immediate upstream suppressor of JP-2 [16]. These structural features of junctions enable regulation of RyR Ca^2+^ release, and they are therefore important modulators of the CICR and determinants of the contractility of heart cells [17, 18].

In failing heart cells, the reduced contractility is attributable at least in part to defective CICR signaling, in which the LCC Ca^2+^ influx cannot trigger sufficient Ca^2+^ release from RyRs. Furthermore, the downregulation of JP-2 caused by elevated miR-24 expression reduced the TT-SR distance, which is the primary mechanism of E-C coupling defects [16, 19–22].

The doxorubicin (DOX)-induced HF model has been used in experimental animal studies [23, 24], and in these models, the cardiotoxicity of DOX causes cardiomyopathy and congestive heart failure [25, 26]. DOX treatment results in the generation of reactive oxygen species (ROS), DNA mutagenesis, cell membrane damage, and apoptosis [27], while excess ROS promote the production of free radicals, leading to mitochondrial damage [28]. In addition, DOX-induced inflammatory injury is involved in the pathogenesis of HF [29].

Few effective cardioprotective drugs are available, and the dose-dependent cardiotoxicity of those in use is an important safety concern. Digoxin, one of the most commonly prescribed drugs for the treatment of HF, has been in use for over two centuries. It prevents DOX-mediated cardiomyopathy by competitively inhibiting the binding of DOX to its receptor [30].

Complementary and alternative medicines may be safe and effective for the treatment of HF. *Lingguizhugan* decoction (LGZG), an ancient Chinese herbal formula from the Treatise on Cold Pathogenic and Miscellaneous Diseases, is used for treating phlegm and fluid retention and for several diseases related to fluid retention [31]. The theory of traditional Chinese medicine holds that spleen Yang deficiency, as well as phlegm and fluid retention, is related to HF [32]. On this basis, LGZG has been used for thousands of years to treat cardiovascular diseases.

LGZG reportedly has hepatoprotective [33–35], anti-obesity, anti-hypertension [36], anti-inflammatory [37], and antioxidant [34] activity in vitro or in vivo, so it may be effective against DOX-induced HF. However, the effect of LGZG on the development of DOX-induced HF and its mechanism of action are unclear.

We investigated whether LGZG protects against HF in rats with DOX-induced HF and explored the underlying mechanism. We focused on the effect of LGZG on the TT-SR junctional structure and its modulation of myocardial contractility.

## Methods

### Preparation of Lingguizhugan decoction

The *Lingguizhugan* decoction consists of the following four Chinese medicines: Poria, Ramulus Cinnamomi, Rhizoma Atractylodis Macrocephalae, and Radix Glycyrrhizae. The full Latin binomial names of the components of *Lingguizhugan* are listed in Table 1. The ratio of the four herbs was 4:3:3:3, and they were obtained from Sichuan Provincial Hospital of Traditional Chinese Medicine. Professor Qinwan Huang of the School of Pharmacy, Chengdu University of Traditional Chinese Medicine, performed micro- and macroscopic authentication of the crude components to ensure that they met the standards of the 2015 Pharmacopoeia of the People’s Republic of China. All voucher specimens were deposited at the College of Basic Medicine, Chengdu University of Traditional Chinese Medicine. High-performance liquid chromatography was performed for quality control of the components of *Lingguizhugan* (Additional file 1: Figure S2).

**Table 1:** Full names of the ingredients of Lingguizhugan

Ingredient of *Lingguizhugan*	Full scientific name	Major identified compounds
Poria	Poria cocos (Schw.) Wolf.	β-pachyman; pachymic acid
Ramulus Cinnamomi	Cinnamomum cassia Presl.	Trans-cinnamic acid
Rhizoma Atractylodis Macrocephalae	Atractylodes macrocephala Koidz.	Atractylenolide I–IV
Radix Glycyrrhizae	Glycyrrhiza uralensis Fisch.	Glycyrrhizic acid

The herbal decoction was prepared as described previously [32]. Briefly, herbal material was placed in a cooking pot containing 500 mL of water and boiled for 30 min, simmered for 20 min, and transferred by filtration. The final volume of the concentrated decoction was 100 mL.

### Chemicals and reagents

DOX was purchased from Sigma (MO, USA; cat. D1515). The primary antibody against JP-2 was obtained from Abcam (Cambridge, UK; cat. ab79071). The B-type natriuretic peptide (BNP) Enzyme-linked Immunosorbent Assay (ELISA) kit was purchased from ZCIBIO (Shanghai, China; cat. ZC-37019), and digoxin was purchased from SINE (Shanghai, China).

### Rats

Adult male Sprague–Dawley rats (180–200 g) were purchased from Chengdu Dashuo Experimental Animal Center. The animals were housed under standardized conditions and received commercial rat chew ad libitum.

### Treatment groups

The rats were randomized into the following six groups: (1) saline intraperitoneal injection plus water intragastrically (control, *n* = 8); (2) DOX intraperitoneal injection plus water intragastrically (DOX); (3) DOX intraperitoneal injection plus digoxin (0.026 mg/kg/d) intragastrically (digoxin, *n* = 8) [38]; (4) DOX intraperitoneal injection plus *Lingguizhugan* decoction (5 mL/kg/d) intragastrically (LD-LGZG, *n* = 8); (5) DOX intraperitoneal injection plus *Lingguizhugan* decoction (10 mL/kg/d) intragastrically group (MD-LGZG, n = 8) [35]; and (6) DOX intraperitoneal injection plus *Lingguizhugan* decoction (15 mL/kg/d) intragastrically (HD-LGZG, n = 8). The rat HF model was established by repeated intraperitoneal injection of DOX [39]. Briefly, DOX (2 mg/kg) in saline was administered intraperitoneally to rats twice a week for 4 weeks (cumulative dose, 16 mg/kg). Beginning on the second day after the final dose of DOX, the indicated treatment was administered orally daily for 4 weeks.

### Sample collection

After treatment for 4 weeks, the rats were euthanized by cervical dislocation under anesthesia induced by intraperitoneal injection of 3% sodium pentobarbital. Blood samples were collected for ELISA, and heart samples were collected for histopathological analysis, transmission electron microscopy (TEM), Western blotting, and quantitative real-time PCR.

### Histopathological analysis

After echocardiography, the heart was removed and cut into two transverse sections. One section was fixed in 4% paraformaldehyde in 0.1 M phosphate-buffered saline overnight and then embedded in paraffin. The other section (5-μm thickness) was stained with hematoxylin and eosin (H&E) as described previously [40].

### Protein preparation and Western blotting

JP-2 expression in cardiac tissue was assessed by Western blotting according to standard protocols. Briefly, protein was extracted from cardiac tissue in radioimmunoprecipitation assay buffer containing a protease inhibitor and centrifuged (12,000 rpm, 10 min, 4 °C). The protein concentration in the supernatant was quantified by bicinchoninic acid assay. Total protein was resolved by sodium dodecyl sulfate–polyacrylamide gel electrophoresis and transferred to polyvinylidene difluoride membranes. The membranes were incubated with an anti-JP-2 antibody (1:1000 dilution) overnight at 4 °C. Images were captured using an ImageQuant LAS4000 Imaging Station (GE), and band densities were quantified using ImageQuant TL software (GE).

### Tem

TEM was performed as described previously [41]. Cardiac tissue was dissected into 1-mm^3^ pieces and fixed in 4% paraformaldehyde and 2% glutaraldehyde in 0.1 M sodium cacodylate buffer (pH 7.2) overnight at 4 °C. Following several washes in buffer, the samples were post-fixed in 2% osmium tetroxide and 1% uranyl acetate for 2 h, rinsed in water, dehydrated in an ascending ethanol series followed by 100% acetone, and infiltrated and embedded in Eponate. Ultrathin sections were cut on a Reichert-Jung microtome (Austria) and mounted onto 200-hex-mesh copper grids. The sections were exposed to the primary stain (5% aqueous uranyl acetate) followed by the secondary stain (lead citrate) and then visualized using a H-600IV TEM. To quantify mitochondrial size and number, eight random fields of view were imaged per group. Mitochondria were identified based on their morphology, and mitochondrial size was measured as the average cross-sectional diameter using Image Pro-Plus 6.0 software.

### Elisa

The level of BNP in serum was measured using a BNP ELISA Kit according to the manufacturer’s instructions.

### Echocardiography

Cardiac function was evaluated non-invasively by M-mode echocardiography as described previously [42]. Briefly, rats were anesthetized with 1% sodium pentobarbital and were fixed in the supine position with the front legs spread. The hairs on the ventral chest and frontal area were removed. Next, ultrasound transmission gel was applied to the precordium. Transthoracic echocardiography was performed using an echocardiograph (Acuson Sequoia model 512, Siemens) equipped with a 25 MHz linear transducer. Also, the left ventricular end-diastolic diameter (LVEDD), left ventricular end-systolic diameter (LVESD), ejection fraction (EF), and fraction shortening (FS) were determined. The sonographer and the analyzer were blinded to the group allocation.

### Determination of the miR-24 and CACNA1S and RyR2 mRNA levels

Total RNA was extracted from cardiac tissue and cells using TRIzol™ reagent (Invitrogen, USA) according to the manufacturer’s instructions. For reverse transcription of miRNA, 1 μg of total RNA was used as the template, together with the Bulge-Loop™ miRNA RT Primer (5 μM) (RIBOBIO, China). For reverse transcription, 1 μg of total RNA was used for synthesis of first-strand cDNA with the iScript™ cDNA Synthesis Kit (Bio-Rad, USA). The mRNA level of CACNA1S and RyR2 was analyzed by quantitative real-time PCR (Bio-Rad) using the iScript™ One-Step RT-PCR Kit with SYBR® Green (Bio-Rad) in a total volume of 20 μL and the gene-specific primers in Additional file 1: Table S1. To assess the expression of miR-24, 10 ng of cDNA product were subjected to real-time PCR amplification using the Bulge-Loop™ miRNA Forward and Reverse Primers (RIBOBIO, China). The thermocycling program was 95 °C for 5 min, followed by 40 cycles of 95 °C for 15 s, 60 °C for 30 s, and 72 °C for 30 s, with a final dissociation step to ensure the specificity of amplification. Each sample was assayed in triplicate. The small nuclear RNA U6 was used as the control for quantification of the miR-24 level, and GAPDH for quantification of the CACNA1S and RyR2 mRNA levels.

### Statistics

Quantitative data are presented as means ± standard error of mean (SEM). Comparisons of multiple groups were determined by one-way ANOVA with Tukey’s post hoc test using SPSS 21.0 (SPSS Inc., Chicago, USA). *P*-values < 0.05 were considered indicative of statistical significance.

## Results

### LGZG improved cardiac function

**Fig. 1:**
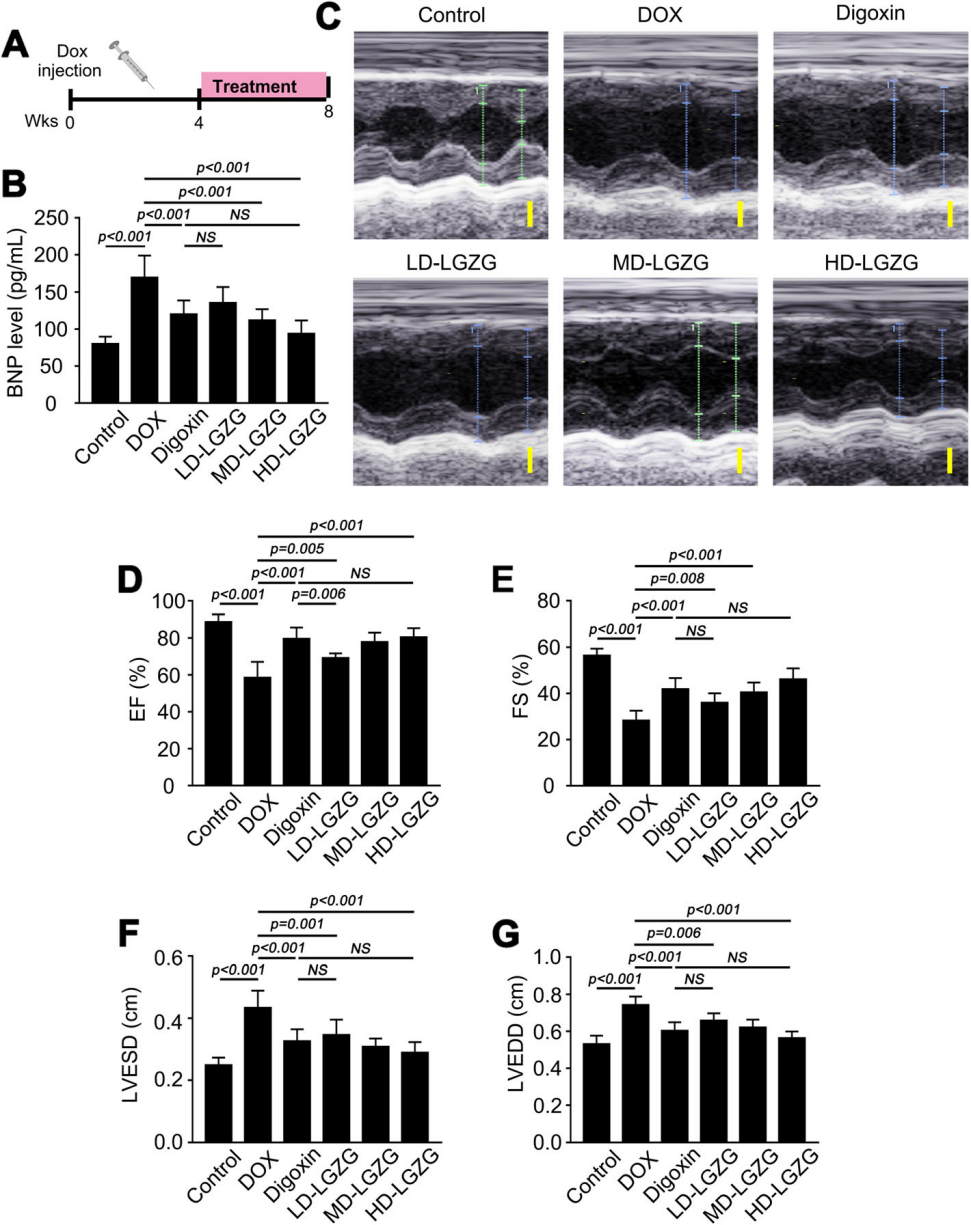
LGZG improved cardiac function. (**a**) Schematic of HF induction by DOX and the treatment protocol. (**b**) BNP level as determined by ELISA. (**c**) Representative echocardiographic images (scale bar, 0.5 cm). Ejection fraction (EF) and fraction shortening (FS), left ventricular end-systolic diameter (LVESD), and left ventricular end-diastolic diameter (LVEDD) are shown in (**d**), (**e**), (**f**), and (**g**), respectively. The experiment was performed in triplicate. Data are mean ± SEM, *n* = 8. The observer was blinded to the group assignment. NS, not significant

**Fig. 2:**
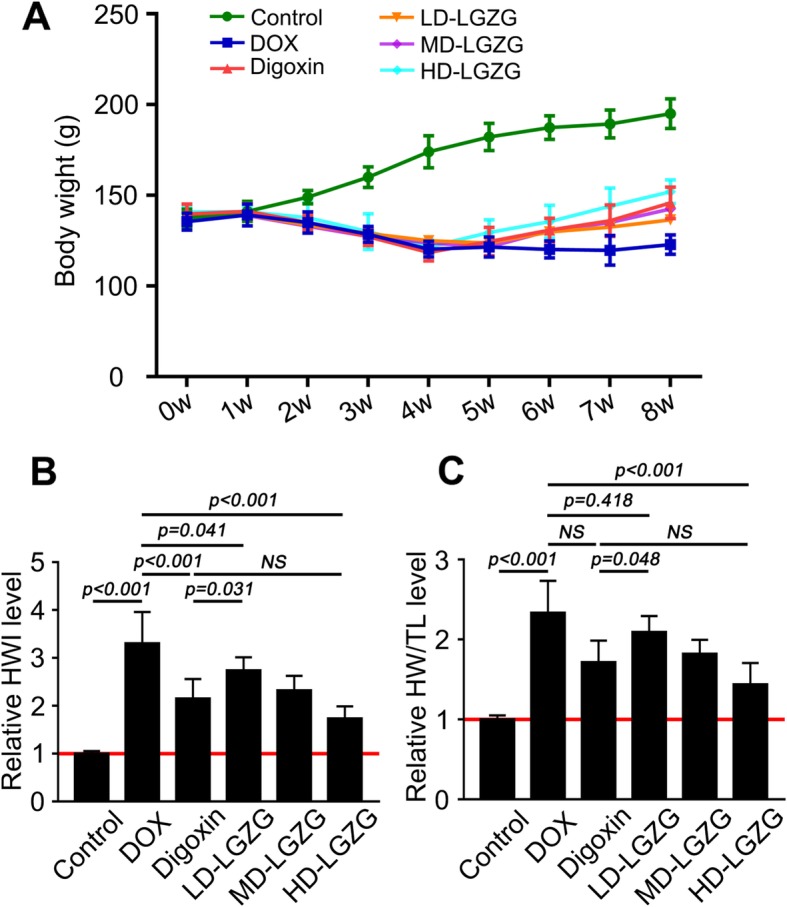
LGZG prevented aggravation of DOX-induced HF. (**a**) Body weight. Effect of LGZGT on HWI and the HW/TL ratio is shown in (**b**) and (**c**), respectively. Values were normalized to the control group. Data are mean ± SEM, n = 8. NS, not significant

To determine the effect of LGZG on HF, we administered DOX (i.p.) to rats for 4 weeks to induce HF. The rats were next treated with saline, digoxin, or LGZG intragastrically for 4 weeks (Fig. 1a). LGZG markedly reduced the serum BNP level in DOX-treated rats (Fig. 1b). Echocardiography was performed to investigate the effect of LGZG on cardiac function in rats with HF (Fig. 1c), and the EF, FS, LVESD, and LVEDD were determined. Cardiac function in DOX-treated rats was improved by LGZG, as indicated by EF (68.89% versus 75.82, 77.87, and 80.45%, respectively) and FS (28.33% versus 36.06, 40.5, and 46.17%, respectively) values (Fig. 1d and e). LGZG reduced LVEDD and LVESD in a dose-dependent manner compared to the model group (Fig. 2f, g), suggesting attenuation of DOX-induced cardiac dilation. Therefore, LGZG improved cardiac function.

### LGZG improved DOX-induced HF

LGZG significantly attenuated cardiac hypertrophy in DOX-induced failing hearts, as indicated by increased body weight (Fig. 2a) and reduced HWI and the HW/TL ratio (Fig. 2b, c), suggesting prevention of further cardiac injury. Therefore, LGZG prevented further aggravation of HF.

### LGZG attenuated the changes in myocardial morphology and ultrastructure

**Fig. 3:**
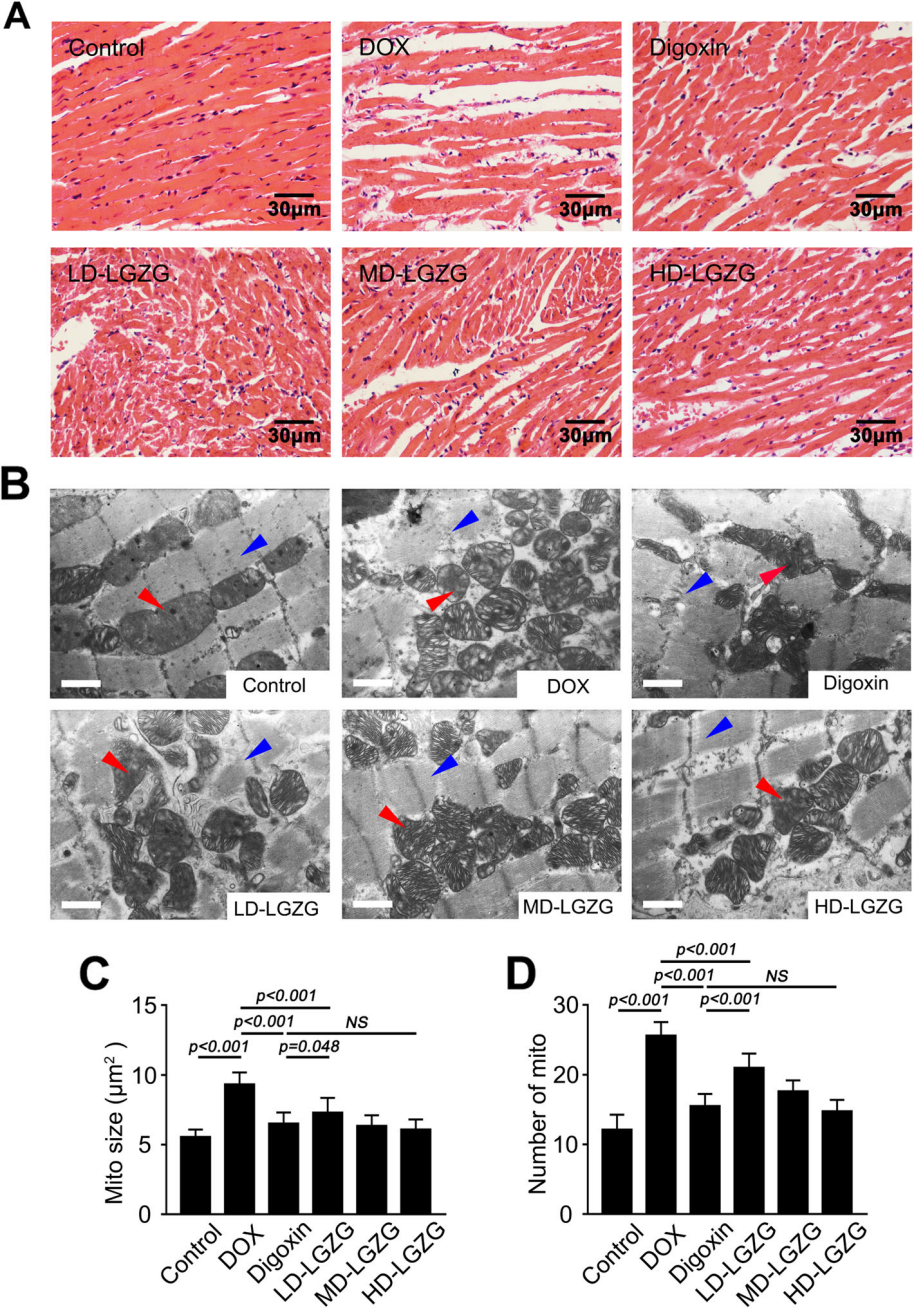
LGZG attenuated the changes in myocardial morphology and ultrastructure. (**a**) Representative images of H&E-stained cardiac tissue. (**b**) Representative TEM images of cardiac tissue (scale bar, 1 μm). Red arrows, mitochondria; blue arrows, myofibrils. Intermyofibrillar mitochondrial size and number are shown in (**c**) and (**d**). Data are mean ± SEM, n = 8. NS, not significant

H&E staining showed that LGZG attenuated myocardial structure disorders of muscle fibers and infiltration of inflammatory cells in DOX-induced HF (Fig. 3a). Also, TEM showed disrupted myocardium and disorganized mitochondria with abnormal cristae structure in the failing heart (Fig. 3b). Cardiac muscle fibers from control rats had a normal myocardial ultrastructure, characterized by laterally aligned myofibrils with highly organized sarcomeres and elongated mitochondria tightly wrapped in strands between the myofibrils. In contrast, fragmented and disrupted myofibrils and disorganized sarcomere arrays were observed in the DOX-treated rats. Extensively fragmented and swollen mitochondria were round or irregular and abnormally agglomerated or dispersed; LGZG reversed these mitochondrial abnormalities. In addition, LGZG decreased the size of swollen mitochondria and suppressed the number of fragmented mitochondria (Fig. 3c and d). Therefore, LGZG blocked DOX-induced changes in cardiac morphology and myocardial ultrastructure.

### LGZG improved TT-SR microstructural remodeling

**Fig. 4:**
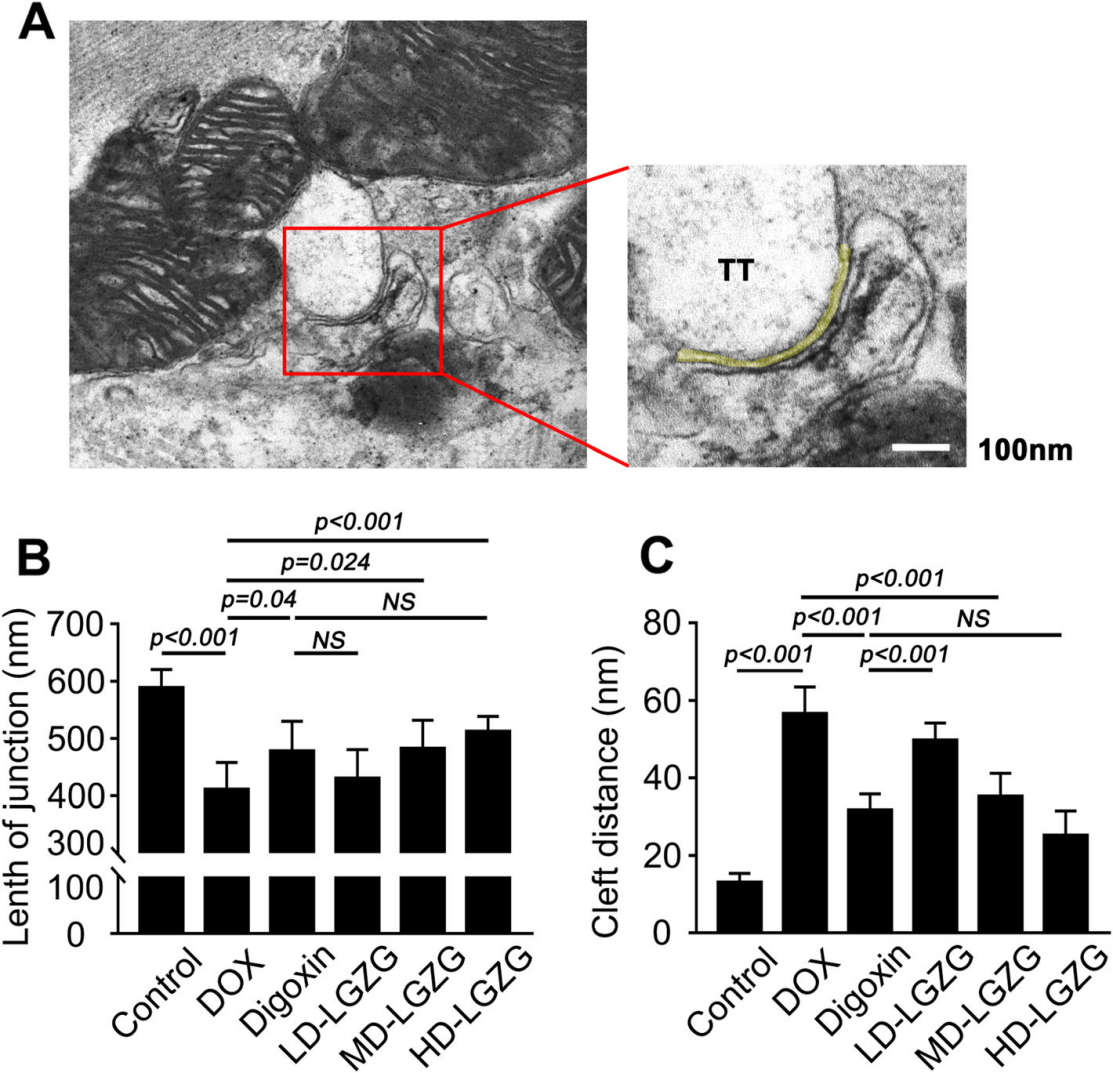
LGZG improved TT-SR microstructural remodeling. (**a**) Representative cardiac TEM images of cardiac tissue from the control group showing selection of a region-of-interest. The junction region (left, red box) was selected and analyzed (right). The TT-SR junctional cleft is marked in yellow. The cleft distance and length of TT-SR junctions were measured as the curvilinear width and length of the yellow line. The average cleft distance and length of junctions are shown in (**b**) and (**c**). Data are mean ± SEM, n = 8. NS, not significant

Cardiomyocyte contraction is controlled by CICR between LCCs in the cell membrane/T-tubules and RyRs in the SR. In addition, TT-SR junctions are remodeled during HF. Based on these reports, the microstructure of TT-SR junctions was observed by TEM (Fig. 4a). Treatment with DOX resulted in a 30% reduction in TT-SR junction length compared to the control. However, different doses of LGZG improved the DOX-induced shortening of TT-SR junctions (5, 17, and 24%, respectively) (Fig. 4b). Similarly, sustained LGZG treatment reduced the DOX-induced increases in the cleft distance of TT-SR junctions (Fig. 4c).

### Mechanism of the effect of LGZG on TT-SR junctions

**Fig. 5:**
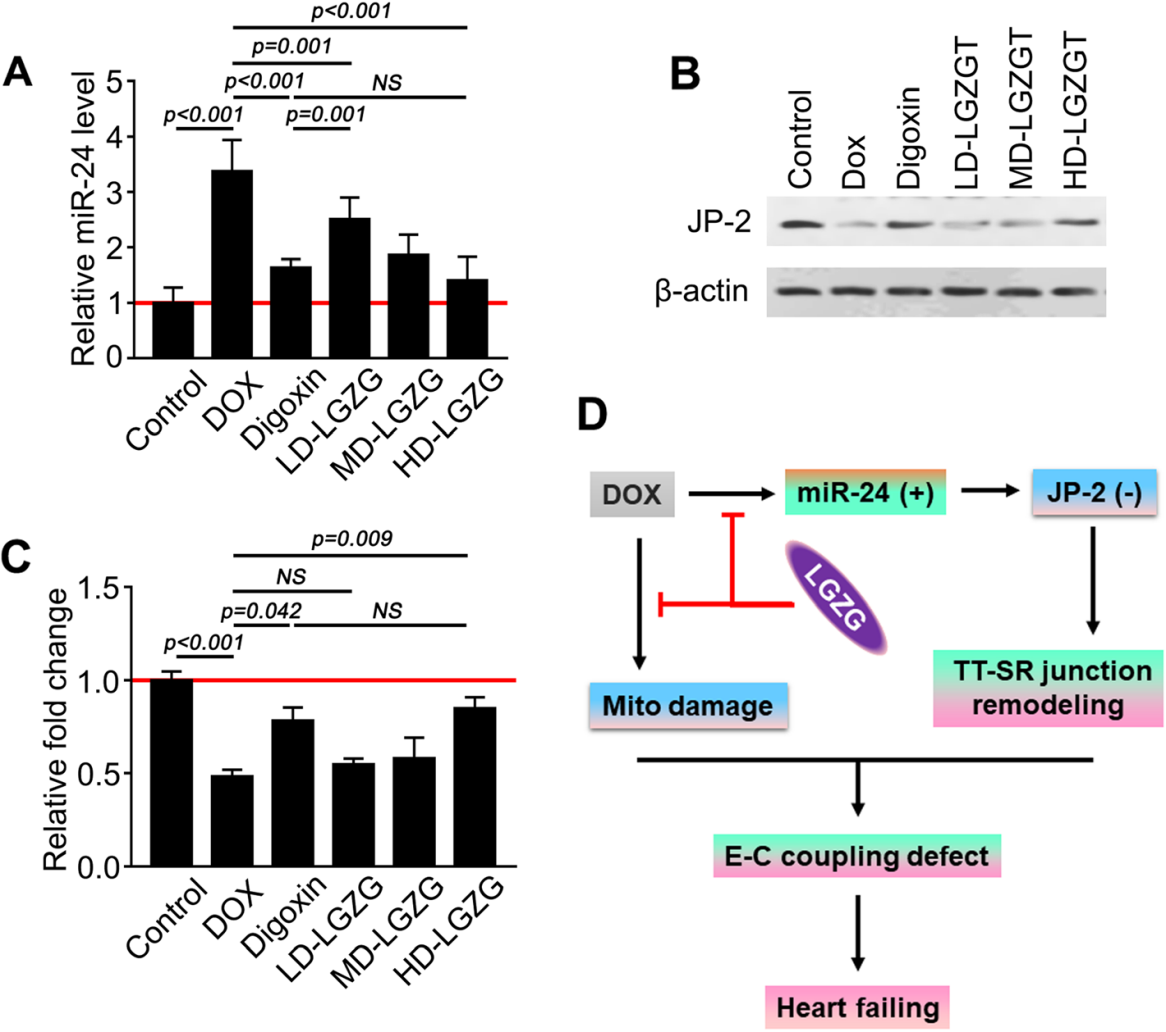
The mechanism by which LGZG regulates TT-SR junctions. (**a**) miR-24 level by quantitative real-time PCR. (**b**) Western blotting and quantification (**c**) of JP-2. Values were normalized to the control group. (**d**) Schematic diagram of the effect of LGZG in the rat model of HF. The experiments were performed in triplicate. Data are mean ± SEM, n = 8. NS, not significant

The LCC-RyR signaling pathway is dysfunctional during HF. Thus, we investigated the effect of LGZG on the LCC-RyR signaling pathway. Quantitative RT-PCR showed that the mRNA levels of CACNA1S and RYR2 were not significantly different after treatment compared with before (Additional file 1: Fig. S1A, B). MiR-24, which is upregulated in HF, is an immediate upstream suppressor of JP-2 [16]. Because miR-24 suppresses JP-2 and inhibits CICR, resulting in LCC-RyR dysfunction, we determined by quantitative RT-PCR whether LGZG promoted JP-2 expression by regulating miR-24. The results showed that miR-24 was upregulated in DOX-treated compared to -untreated hearts (control). However, the level of miR-24 in the LGZG-treated group was markedly lower than that in the DOX group (Fig. 5a). In addition, the decrease in miR-24 level was consistent with the increase in the JP-2 mRNA level (Fig. 4b, c). This suggests that LGZG promotes JP-2 expression by regulating miR-24, which may underlie its effect on HF (Fig. 4d).

## Discussion

Traditional treatments for heart failure include diuretics, followed by angiotensin converting enzyme inhibitors (ACEI) or beta-blockers, and patients with no contraindications can use aldosterone antagonists [43]. Despite advances in therapy, HF remains a major health problem worldwide for which novel therapeutic strategies are needed. Here, we report that LGZG has therapeutic potential for HF.

DOX is an anticancer chemotherapeutic used for solid tumors and acute leukemia. However, the side effects of DOX, especially its cardiotoxicity, limit its utility. DOX causes myocardial architecture and functional abnormalities, including cardiomyocyte hypertrophy and death and increased susceptibility to myocardial infarction, cardiomyopathy, and left ventricular dysfunction. This makes DOX suitable for use in models of non-ischemic cardiomyopathy and HF. Thus, we used a DOX-induced model to evaluate the effect and mechanism of LGZG on HF.

HWI and the HW/TL ratio are used to assess myocardial hypertrophy [44]. As a sensitive marker of cardiac failure, BNP is used for the diagnosis of HF [45], and it reflects not only left ventricular systolic dysfunction but also left ventricular diastolic dysfunction and right ventricular dysfunction. Echocardiography is a versatile, noninvasive tool for measuring cardiac function and structure. Our data are consistent with previous reports that DOX induced signs of cardiomyopathy in the form of increased HWI, HW/TL ratio, and BNP values, indicating severe cardiac dysfunction. Interestingly, we found that LGZG at clinical doses significantly attenuated HWI, the HW/TL ratio, and abnormal BNP levels in a dose-dependent manner. Also, LGZG reduced LVEDD and LVESD and increased EF and FS, suggesting prevention of DOX-induced deterioration of cardiac function.

Various ultrastructural changes occur in DOX-associated HF, including loss of myofibrils, disarray of sarcomere structure, dilation of the sarcoplasmic reticulum, and swelling of mitochondria [46–48]. Thus, our histopathological and TEM data revealed structural disorders of myocardial fibers, infiltration of inflammatory cells, fragmented and disrupted myofibrils, and disorganized sarcomere arrays. These ultrastructural changes in the failing heart were significantly attenuated by LGZG.

Cardiac contractility and relaxation are determined by the Ca^2+^ cycle, which is critical for the mobilization of intracellular Ca^2+^ in E-C coupling. The Ca^2+^-triggering concentration generated by LCCs decays by several orders of magnitude with distance [49, 50]; therefore, the CICR response is highly dependent on the distance between LCCs and RyRs. Several models have been proposed to explain the decreased EC coupling gain: mismatch in LCC and RyR locations, increased gap between SR and TT membranes, orphaned RyRs due to TT reorganization, and a decrease in size and/or shift in position [22]. Consistent with these findings, we found that the size (length and width) of the TT-SR junction was reduced in failing cardiomyocytes; this was significantly ameliorated by LGZG.

JP-2, a key regulator of TT-SR junctions, is downregulated or mislocalized in all animal models of HF [19, 21] and in patients with HF. miR-24 is a direct regulator of JP-2. The high expression level of miR-24 in the failing heart suppresses JP-2 expression [16, 51, 52]. Consistent with these findings, we observed a significant decrease in the expression of JP-2 and a concomitant increase in that of miR-24 in cardiac tissue in the DOX group; these effects were reversed by LGZG.

Although we showed that LGZG regulates miRNA and JP-2 expression and improves cardiac function, we did not demonstrate a direct effect of LGZG on these factors. In addition, traditional Chinese medicines are multi-component and multi-target, so the active components need to be identified. Interestingly, digoxin showed a similar effect to LGZG. The cardiotonic effect of digoxin is attributed to cytoplasmic Na^+^ accumulation, which induces Ca^2+^ influx by reverse sodium–calcium exchange (NCX) [53, 54]. Therefore, we suspect that by improving the calcium ion pathway, promoting myocardial contractility, and ultimately regulating the expression of miR-24 and JP-2 by feedback may be the mode of action of digoxin. However, further investigation is needed.

## Conclusions

Taken together, our findings demonstrate that LGZG inhibits miR-24 expression and promotes that of JP-2, improving TT-SR microstructural remodeling and attenuating DOX-induced HF. These results suggest the factors targeted by LGZG to ameliorate HF, and they provide experimental evidence for LGZG treatment of related diseases.

## Supplementary information

**Additional file 1: Fig. S1.** LGZG did not affect the mRNA level of CACNA1S and RyR2. (A) RyR2 and (B) CACNA1S mRNA level by quantitative real-time PCR. The experiments were performed in triplicate. Data are mean ± SEM, *n* = 8. NS, not significant. **Fig. S2.** HPLC for quality control of the components of LGZG. (A) The representative ingredients of LGZG include pachymic acid, trans-cinnamic acid, atractylenolide I, glycyrrhizic acid, which are used for quality control. (B) HPLC was preform to analysis of LGZG. **Table S1.** Primer sets used for real-time PCR.

## Data Availability

The datasets used and/or analysed during the current study are available from the corresponding author on reasonable request.

## Abbreviations

ACEI: Angiotensin converting enzyme inhibitors
BNP: B-type natriuretic peptide
CICR: Ca^2+^-induced Ca^2+^ release
CVD: Cardiovascular disease
DOX: Doxorubicin
E-C: Excitation-contraction
EF: Ejection fraction
FS: Fraction shortening
HF: Heart failure
HW/TL: Heart weight/tibia length
HWI: Heart weight index
JP-2: Junctophilin-2
LCCs: L-type Ca^2+^ channels
LGZG: Lingguizhugan decoction
LVEDD: Left ventricular end-diastolic diameter
LVESD: Left ventricular end-systolic diameter
miR-24: MicroRNA-24
NCX: Sodium-calcium exchange
ROS: Reactive oxygen species
RyRs: Ryanodine receptors
TEM: transmission electron microscopy
TT-SR: T-tubule-sarcoplasmic reticulum
