# Supplementary material for: Lingguizhugan decoction attenuates doxorubicin-induced heart failure in rats by improving TT-SR microstructural remodeling
Source: BMC Complement Altern Med. 2019 Dec 11;19:360. doi: 10.1186/s12906-019-2771-6 (PMC6907350; doi:10.1186/s12906-019-2771-6)
Supplement: Supplementary file 1 — Additional file 1: Fig. S1. LGZG did not affect the mRNA level of CACNA1S and RyR2. (A) RyR2 and (B) CACNA1S mRNA level by quantitative real-time PCR. The experiments were performed in triplicate. Data are mean ± SEM, n = 8. NS, not significant. Fig. S2. HPLC for quality control of the components of LGZG. (A) The representative ingredients of LGZG include pachymic acid, trans-cinnamic acid, atractylenolide I, glycyrrhizic acid, which are used for quality control. (B) HPLC was preform to analysis of LGZG. Table S1. Primer sets used for real-time PCR. [file 12906_2019_2771_MOESM1_ESM.pdf]

Supplementary figure 1

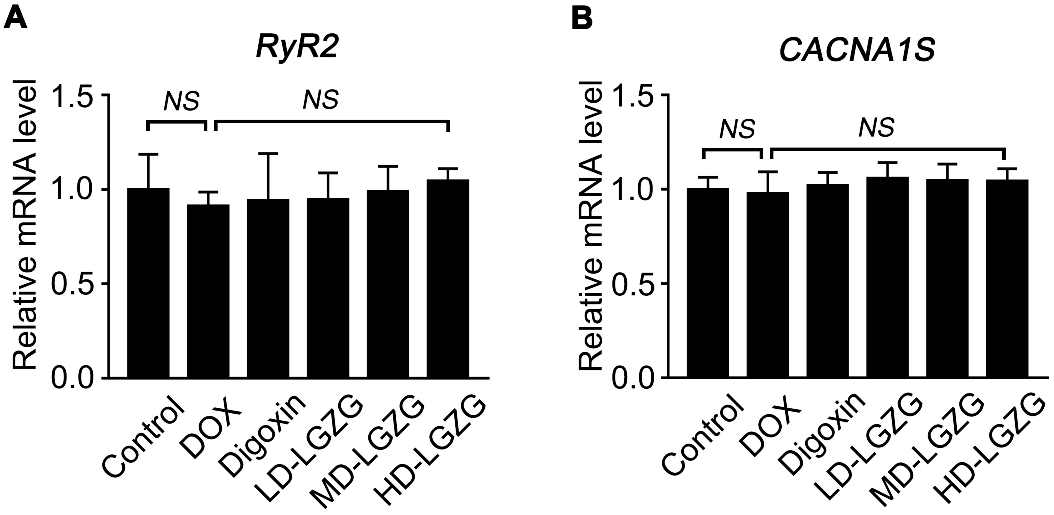

Supplementray figure 2

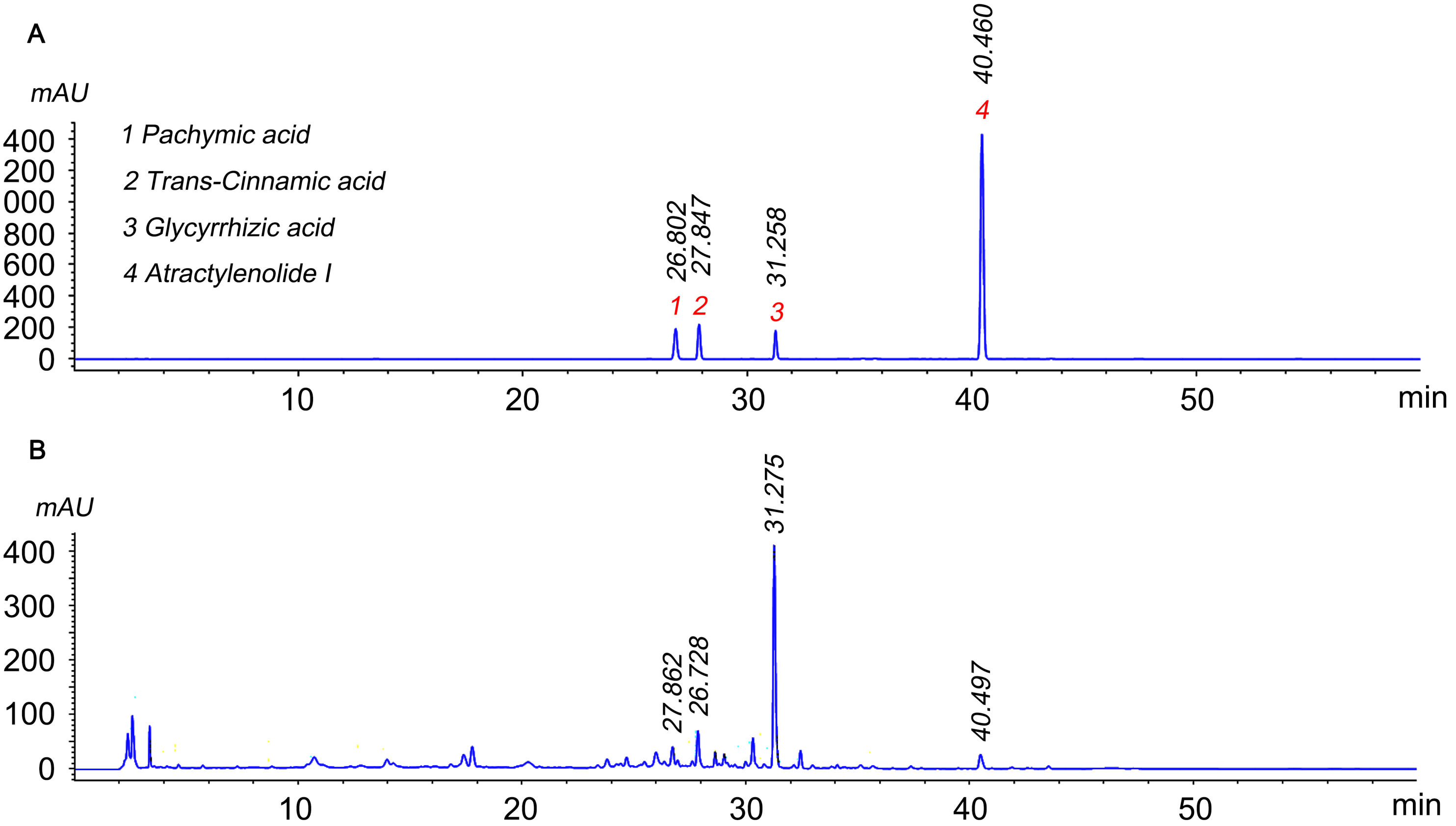

Supplementary Table 1. Primer sets used for real-time PCR.

Gene	Sense Primer (5′ - 3′)	Antisense Primer (5′ - 3′)
RyR2	GGCTTCTTCCGCATTGTCTC	TCTCCTCGCACCTCATCCT
CACNA1S	ACAACCAGTCGGAACAGATGA	TCGGAACAGGCGGAAGAAG
GAPDH	GGCAAGTTCAACGGCACA	TCTCGCTCCTGGAAGATGG

Uncropped western blots

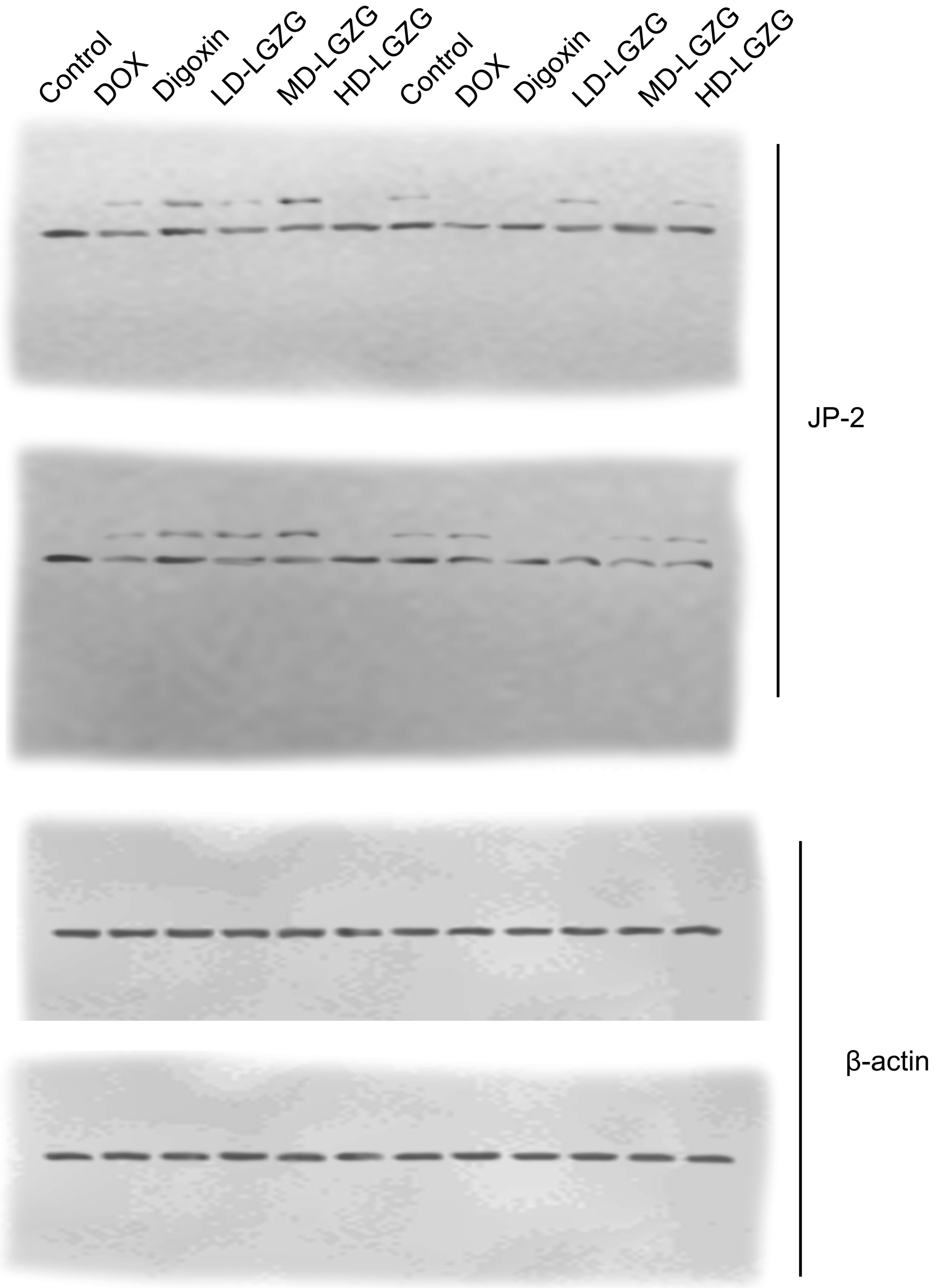

		Pixels of bot		
		**JP2**	**β-actin**	JP2:β-actin
	CON	87606	97111	0.902122314
	MODEL	53217	104395	0.509765793
	DIGOXIN	72304	105856	0.683041112
**band1-1**	LD-LGZG	49638	94223	0.526814048
	MD-LGZG	52907	102387	0.516735523
	HD-LGZG	72095	82797	0.870744109
	CON	72022	83751	0.859953911
	MODEL	40060	89628	0.44695854
	DIGOXIN	61211	84490	0.724476269
**band1-2**	LD-LGZG	50190	89488	0.560857322
	MD-LGZG	68388	84793	0.806528841
	HD-LGZG	66992	89532	0.748246437
		JP2-2	actin2	
	CON	82415	79672	1.034428657
	MODEL	30152	85325	0.35337826
	DIGOXIN	69219	79034	0.875812941
**band2-1**	LD-LGZG	38050	87605	0.43433594
	MD-LGZG	38811	84517	0.45920939
	HD-LGZG	60946	72442	0.841307529
	CON	65666	78114	0.840643163
	MODEL	41697	92854	0.449059814
	DIGOXIN	45139	79732	0.566134049
**band2-2**	LD-LGZG	37155	78410	0.473855376
	MD-LGZG	25121	76290	0.329282999
	HD-LGZG	45714	72503	0.630511841

Supplementary materials of RT-PCR raw data of U6 and miR-24

	Ct of gene		Ct average		$^{\triangle}CT=CT_{miR-24}-Ct_{u6}$		$^{\triangle\triangle}CT$	$2^{-\triangle\triangle CT}$
Groups	U6	miR-24	U6	miR-24				
control-1	15.62	21.45						
	15.61	21.45	15.62	21.50	5.88		-0.23	1.17
	15.64	21.60						
control-2	15.37	21.51						
	15.46	21.37	15.48	21.37	5.89		-0.22	1.16
	15.62	21.24						
control-3	15.80	22.06						
	15.83	22.01	15.88	22.04	6.16		0.05	0.96
	16.00	22.04						
control-4	16.42	22.72						
	16.52	22.59	16.50	22.63	6.13		0.02	0.99
	16.57	22.58						
control-5	16.08	21.55						
	16.16	21.68	16.10	21.53	5.43		-0.67	1.59
	16.05	21.36						
control-6	15.88	22.24						
	15.76	22.24	15.69	22.34	6.65		0.54	0.69
	15.42	22.53						

control-7	15.54	21.68						
	15.27	21.20	15.33	21.38	6.05		-0.05	1.04
	15.17	21.26						
control-8	15.06	21.72						
	14.97	21.71	15.05	21.71	6.66	6.11	0.55	0.68
	15.11	21.69						
DOX-1	17.06	21.36						
	16.85	21.03	16.72	21.12	4.40		-1.71	3.26
	16.24	20.96						
DOX-2	16.04	20.73						
	15.88	20.63	15.83	20.68	4.85		-1.26	2.39
	15.56	20.67						
DOX-3	17.42	21.02						
	17.34	20.72	17.26	20.81	3.55		-2.55	5.87
	17.01	20.69						
DOX-4	16.97	21.08						
	16.87	20.97	16.95	21.04	4.08		-2.02	4.06
	17.02	21.06						
DOX-5	17.15	21.75						
	17.08	21.72	17.09	21.77	4.67		-1.43	2.70
	17.05	21.83						
DOX-6	15.59	20.45						

	15.87	20.46	15.76	20.37	4.62		-1.49	2.81
	15.81	20.21						
DOX-7	16.99	20.78						
	17.04	20.79	16.96	20.92	3.96		-2.15	4.44
	16.86	21.19						
DOX-8	18.90	23.85						
	18.88	23.67	18.87	23.69	4.82		-1.28	2.43
	18.82	23.55						
Digoxin-1	14.94	22.06						
	14.73	21.97	14.81	21.94	7.13		1.02	0.49
	14.77	21.80						
Digoxin-2	15.21	21.66						
	15.15	21.54	15.23	21.62	6.39		0.29	0.82
	15.32	21.66						
Digoxin-3	14.11	20.91						
	13.99	20.80	14.04	20.79	6.75		0.64	0.64
	14.03	20.66						
Digoxin-4	15.02	19.74						
	14.92	19.64	14.89	19.63	4.74		-1.37	2.58
	14.73	19.51						
Digoxin-5	15.32	21.73						
	15.51	21.56	15.5	21.56	5.98		-0.13	1.09

			8					
	15.91	21.39						
	15.49	21.33						
Digoxin-6	15.21	21.15	15.31	21.18	5.87		-0.23	1.18
	15.23	21.07						
	15.41	20.35						
Digoxin-7	15.05	20.02	15.20	20.14	4.95		-1.16	2.23
	15.13	20.06						
	15.71	21.52						
Digoxin-8	15.51	21.57	15.53	21.59	6.07		-0.04	1.03
	15.36	21.69						
	17.13	23.02						
LD-LGZG-1	17.31	23.07	17.22	23.03	5.81		-0.30	1.23
	17.23	23.00						
	17.12	23.19						
LD-LGZG-2	17.25	23.10	17.18	23.17	5.99		-0.12	1.08
	17.17	23.22						
	16.67	21.97						
LD-LGZG-3	16.79	21.90	16.76	21.88	5.12		-0.99	1.98
	16.82	21.77						
	15.46	21.48						
LD-LGZG-4	15.78	21.68	15.72	21.59	5.87		-0.23	1.18

	15.91	21.61						
LD-LGZG-5	15.92	22.47						
	16.36	22.62	16.15	22.51	6.37		0.26	0.83
	16.16	22.45						
LD-LGZG-6	17.29	22.18						
	17.01	21.88	17.16	21.99	4.83		-1.28	2.42
	17.19	21.92						
LD-LGZG-7	17.16	21.60						
	16.92	21.12	17.08	21.28	4.20		-1.91	3.76
	17.17	21.12						
LD-LGZG-8	17.12	21.78						
	17.31	21.47	17.31	21.63	4.32		-1.78	3.44
	17.50	21.65						
MD-LGZG-1	17.05	23.24						
	17.04	23.08	16.86	23.04	6.18		0.07	0.95
	16.49	22.80						
MD-LGZG-2	15.57	21.94						
	15.71	21.68	15.71	21.72	6.01		-0.10	1.07
	15.84	21.53						
MD-LGZG-3	16.32	22.04						
	16.31	21.97	16.23	21.96	5.73		-0.38	1.30
	16.06	21.86						

MD-LGZG-4	15.81	20.57						
	15.89	20.53	15.81	20.61	4.80		-1.31	2.48
	15.74	20.73						
MD-LGZG-5	16.10	20.70						
	16.20	20.72	16.18	20.77	4.59		-1.52	2.86
	16.25	20.90						
MD-LGZG-6	15.42	20.66						
	15.54	20.87	15.58	20.84	5.26		-0.84	1.79
	15.78	21.00						
MD-LGZG-7	15.61	21.05						
	15.65	20.98	15.67	21.08	5.41		-0.69	1.62
	15.74	21.21						
MD-LGZG-8	15.12	21.08						
	15.32	21.06	15.28	21.01	5.73		-0.37	1.29
	15.40	20.90						
HD-LGZG-1	16.38	22.62						
	16.35	22.60	16.39	22.67	6.28		0.17	0.89
	16.43	22.78						
HD-LGZG-2	15.05	20.53						
	15.15	20.40	15.11	20.50	5.39		-0.72	1.64
	15.12	20.56						
HD-	16.42	21.05						

LGZG-3	16.53	20.94	16.54	21.04	4.50		-1.61	3.04
	16.66	21.12						
HD-LGZG-4	15.98	22.76						
	15.73	22.65	15.92	22.76	6.84		0.73	0.60
	16.06	22.87						
HD-LGZG-5	16.76	22.21						
	16.52	22.07	16.75	22.23	5.48		-0.62	1.54
	16.96	22.41						
HD-LGZG-6	15.25	21.80						
	15.07	21.61	15.18	21.70	6.51		0.41	0.75
	15.23	21.68						
HD-LGZG-7	16.84	22.92						
	16.94	22.92	16.87	22.94	6.07		-0.04	1.03
	16.83	22.97						
HD-LGZG-8	15.33	20.28						
	15.20	20.23	15.33	20.31	4.98		-1.12	2.18
	15.46	20.43						

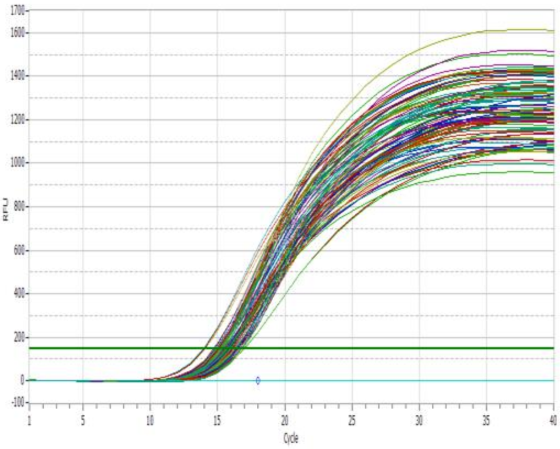

Fig. 1-1 Amplification curve of U6 (1)

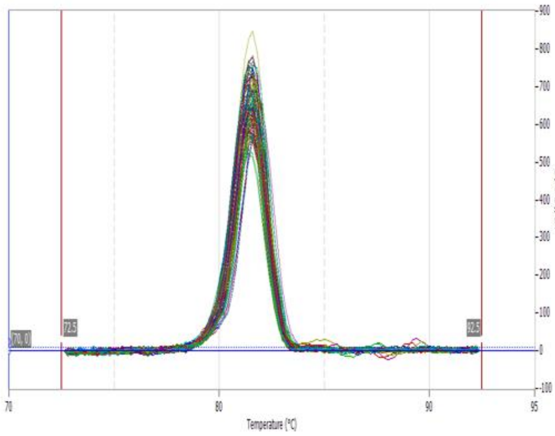

Fig 1-2 Melting curve of U6 (1)

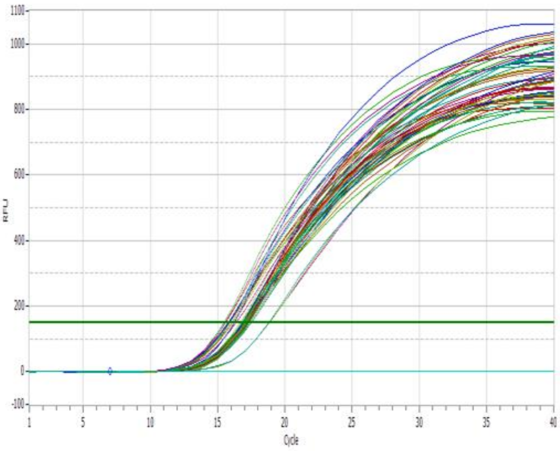

Fig. 1-3 Amplification curve of U6 (2)

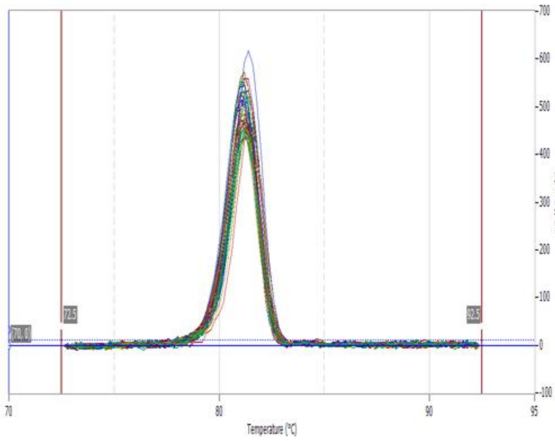

Fig. 1-4 Melting curve of U6 (2)

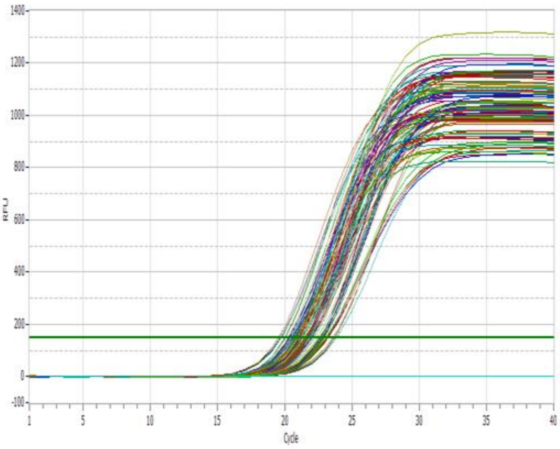

Fig. 1-5 Amplification curve of miR-24 (1)

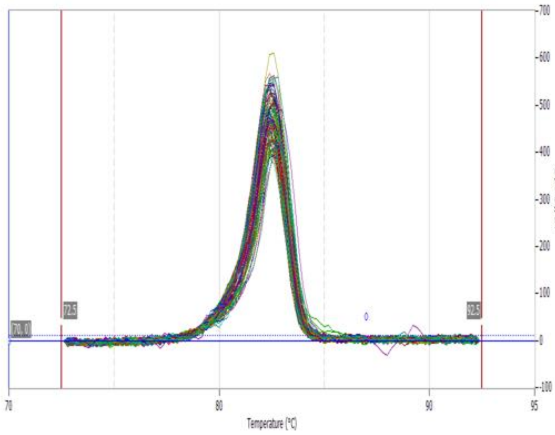

Fig. 1-6 Melting curve of miR-24 (1)

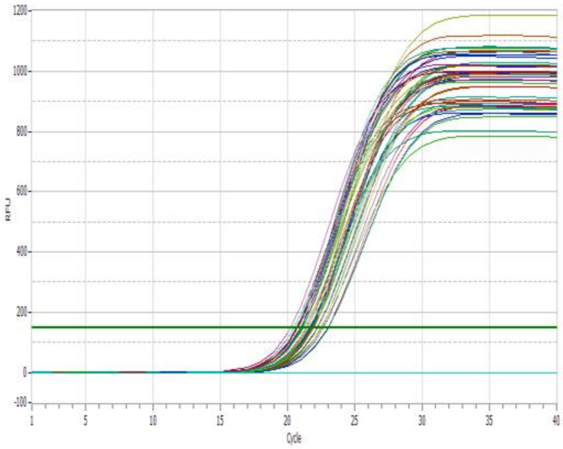

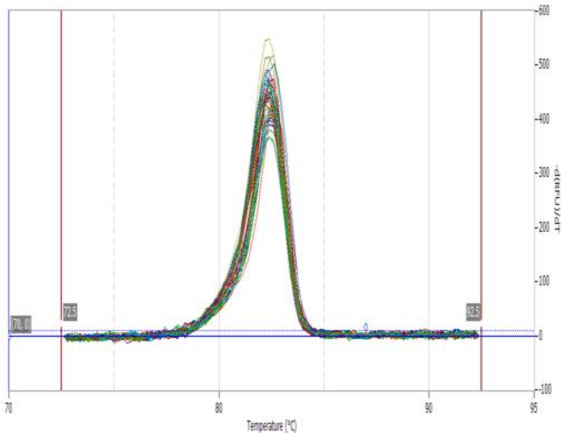

Fig. 1-7 Amplification curve of miR-24 (2)	Fig. 1-8 Melting curve of miR-24 (2)
